# Gene expression profiling identifies Fibronectin 1 and CXCL9 as candidate biomarkers for breast cancer screening

**DOI:** 10.1038/sj.bjc.6605511

**Published:** 2010-01-12

**Authors:** E Ruiz-Garcia, V Scott, C Machavoine, J M Bidart, L Lacroix, S Delaloge, F Andre

**Affiliations:** 1 Breast Cancer Translational Research Unit, UPRES EA03535, Université Paris XI, Institut Gustave Roussy, Villejuif, France; 2 Department of Laboratory Medicine, Institut Gustave Roussy, Villejuif, France; 3 Breast Cancer Unit, Department of Medicine, Institut Gustave Roussy, Villejuif, France

**Keywords:** breast cancer, DNA arrays, screening, biomaker

## Abstract

**Background:**

There is a need to develop blood-based bioassays for breast cancer (BC) screening. In this study, differential gene expression between BC samples and benign tumours was used to identify candidate biomarkers for blood-based screening.

**Methods:**

We identified two proteins (Fibronectin 1 and CXCL9) from a gene expression data set that included 120 BC samples and 45 benign lesions. These proteins fulfil the following criteria: differential gene expression between cancer and benign lesion, protein released in the extracellular medium and stable in the serum, commercially available ELISA kit, ELISA accuracy in a feasibility study. Protein concentrations were determined by ELISA. Blood samples were from normal volunteers (*n*=119) and early BC patients (*n*=133).

**Results:**

Seventy-three per cent of patients had cT1-T2 tumour. Patients had higher CXCL9 and Fibronectin 1 concentrations than volunteers. CXCL9 mean concentration was 851 and 635 pg ml^−1^ for patients and volunteers respectively (*P*=0.013). CXCL9 concentration was significantly higher in patients with estrogen receptor (ER)-negative compared with volunteers (*P*=0.003), data consistent with gene expression profile. Fibronectin 1 mean concentration was 190 *μ*g ml^−1^ for patients and 125 *μ*g ml^−1^ for volunteers (*P*<0.001). Areas under the curve for BC diagnosis were 0.78 and 0.62 for Fibronectin 1 and CXCL9 respectively. A combined score including Fibronectin 1 and CXCL9 dosages presented 53% of sensitivity and 98% of specificity. Similar performances were observed for ER-negative tumours.

**Conclusions:**

This study suggests that Fibronectin 1/CXCL9 dosage in serum could screen a significant rate of BC, including ER-negative, and that differential gene expression analysis is a good approach to select candidate biomarkers to set up blood assays cancer screening.

Breast cancer (BC) is the most frequent cancer in women. World estimation is 850 000 new cases per year with 340 000 deaths per year. In 2005, France had reported 49 814 new cases. Although several advances (mammogram screening and post-operative treatments) have allowed improvements in 5-year survival in this disease, more than 11 000 women died of BC in France (La situation du cancer en France). Breast cancer incidence in the European population in 2006 was 29%, with a mortality rate of 18% (Karim-Kos *et al*, 2008). Early diagnosis could decrease BC mortality by means of cancer downstaging. As an illustration, randomised studies have shown that mammogram screening decreases the BC death rates by 21% (Nystrom *et al*, 2002). Although mammogram screening has allowed an undisputable benefit, it has several limitations. First, it is associated with a significant rate of false-positive results, leading to unnecessary biopsies or surgeries (Fletcher and Elmore, 2003; Delaloge *et al*, 2005). Second, this technology mainly detects slowly proliferating tumours that occur after the age of 50 years, but exhibits poor performance to screen aggressive tumours, especially in young women in whom breast density is high. Finally, although mass screening by mammogram has been well implemented in most of the European countries, 20% of the population are still not compliant with it (Hakama *et al*, 2008). These patients usually present poor income and are excluded from this medical advance. Based on the observation that a mammogram offers a significant, but suboptimal, screening for BC, new approaches have been tested to improve BC detection. New radiological technologies have been tested in this setting. MRI has been shown to detect most of the cancers early, including those in younger women, but this approach is limited by a high rate of false-positive results, together with a high cost. Ultrasonography, combined with a mammogram, also increases the likelihood of BC detection, but is once again associated with high rate of false positivity.

Until now, no serum biomarker has been shown to allow an early diagnosis of BC. As a consequence, current ASCO guidelines do not recommend the use of serum biomarker (CA15-3, CEA) for BC screening (Harris *et al*, 2007).

These data point out the need to develop new easy-to-do tests that would improve BC detection and decrease the rate of false-positive results associated with radiological examinations. In this study, we aimed at generating a blood-based assay that could screen BC. To achieve this goal, we first identified genes overexpressed in BC compared with benign lesions. We then determined which of these genes encoded for proteins released in the extracellular medium. Finally, we performed dosage of these proteins in the blood of overall 252 BC or normal volunteers to determine whether these proteins were more concentrated in patients with BC.

## Patients and methods

### Identification of candidate proteins for breast cancer screening

**Figure 1:**
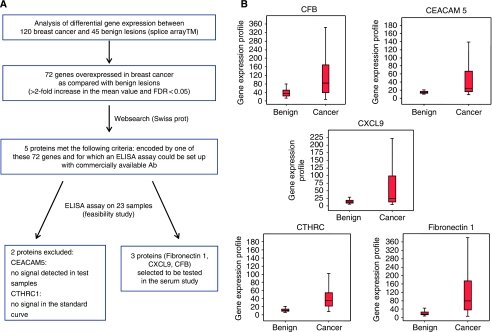
(**A**) Selection of the three candidate proteins for analyses of serum samples. (**B**) Gene expression levels for the five candidate genes in cancer and benign lesions.

To identify which genes were overexpressed in BC, we used a public data set of exon arrays (Andre *et al*, 2009). This data set includes 120 BC and 45 benign lesions. Samples were obtained by fine needle aspiration of breast lesions. Gene expression levels were determined as previously reported. The strategy used to select candidate biomarkers for blood-based screening is reported in Figure 1A. Genes overexpressed in BC (>2-fold increase and FDR<0.05) that encode for a protein released in the extracellular medium were selected for further analyses. To determine which genes encoded for a protein released in extracellular medium, we looked at the protein location in UniProt Knowledgebase (UniProtKB) section Swiss-Prot, to obtain functional information on proteins (http://www.uniprot.org/). Nevertheless, other methods of protein identification could be used. For example, we could use a difference between including Signal P and TMHMM databases. Signal P ((http://www.cbs.dtu.dk/services/SignalP/)) and TMHMM ((http://www.cbs.dtu.dk/services/TMHMM/) recognise signal peptides at the N-terminal and transmembrane regions respectively. Proteins predicted by Signal P but not TMHMM could be secreted in the serum, and therefore represent a potential target for ELISA.

Of the proteins released in extracellular medium, we only kept for further analyses those for which an ELISA could be set up using commercially available antibodies. This approach allowed us to identify five candidate proteins (Fibronectin 1, CXCL9, CEACAM5, CHTRC1 and Complement factor B). Of these five, Complement Factor B (CFB) will no longer be considered, as the protein is unstable in serum.

### Patient selection

Concentrations of candidate biomarkers were assessed in serum samples from 133 women with breast adenocarcinoma and 119 healthy women. Breast cancer patients were retrospectively selected to have presented a primary BC without metastases at diagnosis between 1999 and 2006. Blood samples were obtained at the time of diagnosis. Normal volunteers were healthy blood donors. They presented neither fever, nor cancer history, nor chronic infectious disease (HCV, HBV, HIV) history. As normal volunteers were aged between 18 and 62 years, we focused the analysis of samples from cancer patients in women aged between 18 and 60 years.

### Sample processing

Archived serum specimens were obtained from the Biological Resource Center of Gustave Roussy Institute. All samples have consent in accordance with approval granted by the ethics committee of the medical centre. Control sera were obtained in 2008 with a signed informed consent from normal volunteers of the French Blood Institution. All samples were centrifuged and aliquoted after collection and stored at −80 °C until the assays were performed. No repeat freezing or thawing was permitted.

### ELISA

Three proteins were measured with commercially available enzyme immunoassay kits. Human MIG ELISA set BD OptEIA (Ref. 550998; BD Biosciences–Pharmingen, Franklin Lakes, NJ, USA) was used to determine CXCL9 concentration. Fibronectin 1 was measured with Human Fibronectin ELISA BMS2028 (Bender MedSystems, San Diego, CA, USA). Carcinoembryonic antigen-related cell adhesion molecule 5 (CEACAM5) concentrations were assessed using Carcinoembryonic Antigen Enzyme Immunoassay Test Kit (catalog no. 07BC-1011; MP Biomedicals, Solon, OH, USA). All the measurements were performed following the manufacturer's instructions.

As no ELISA was commercially available for CHTRC1, the ELISAs were set up in house (not shown). Nevertheless, positive controls were not detected for CHTRC1. This latter biomarker was not further explored in this study.

Absorbance was read at 450 nm using a spectrophotometer (ELx808; Fisher Bioblock Scientific, Pittsburgh, PA, USA). Sera concentration of each protein was interpolated from a standard curve, which was generated using the respective purified or recombinant protein.

ELISAs were first applied to a set of 23 blood samples that included 14 BC patients. If a protein was detected, the ELISA was then performed in the study population (*n*=252).

### Statistical analyses

Statistical significance was determined with Student's *t*-test (two tailed) comparison between two groups of data sets. A *P*-value <0.05 was considered significant. The area under an ROC curve (AUC) is a measure of the overall discriminatory power of the marker (with AUC 1.0 corresponding to perfect prediction and AUC 0.50 corresponding to no discrimination) and was calculated with SPSS software version 15.0 (Chicago, IL, USA).

## Results

### Selection of candidate biomarkers for analyses of serum samples

The process for selection of candidate proteins is reported in Figure 1A. We first identified candidate genes in a data set of exon expression arrays that included 120 BCs and 45 benign lesions (Andre *et al*, 2009). Gene expression levels were defined as previously described. Of the 72 genes that presented more than a twofold increase in cancer compared with benign lesions, 5 were selected to encode for a protein detectable by ELISA and released in the extracellular medium (Swiss-Prot). These five genes include Fibronectin 1, MIG1 (CXCL9), CFB, Collagen triple helix repeat containing 1 (CTHRC1) and CEACAM5. Figure 1B reports the gene expression levels for these five candidate genes in malignant and benign breast lesions. We then assessed whether these five candidate genes were differentially expressed according to estrogen receptor (ER) expression. Fibronectin 1 and CFB were overexpressed in ER-positive compared with ER-negative disease (*P*=0.02 and 0.004 respectively, *t*-test). Conversely, MIG1 was overexpressed in ER-negative compared with ER-positive disease (*P*=0.05, *t*-test; data not shown).

We then assessed the feasibility of ELISA for the five candidate proteins. CEACAM5 was not detectable in the 23 samples tested. In addition, it was not possible to obtain any signal with recombinant protein (positive control) for CTHRC1, although ELISA provided a signal in some of the 23 samples. For CFB, Fibronectin 1 and CXCL9, standard curve provided a consistent signal and protein was detectable in at least 1 of the 23 samples. As CFB is unstable, only CXCL9 and Fibronectin 1 will therefore be analysed.

### Patient characteristics for the serum biomarker study

The serum biomarker study was performed on 252 samples from either normal volunteers (*n*=119) or women with non-metastatic breast adenocarcinoma (*n*=133). Samples were obtained between diagnosis and first treatment for all the patients. Patient characteristics are reported in Table 1. Briefly, 73% of the patients presented a cT1-T2 tumour; 34% and 22% of the tumours were ER-negative and Her2+++ respectively. CA15-3 concentration was assessed in 133 patients. Only 14% of these patients presented an elevation of CA15-3 (⩾30 IU ml^−1^), suggesting that conventional serum markers could not screen patients in this study population.

### Fibronectin 1 serum concentration in cancer patients *vs* normal volunteers

**Figure 2:**
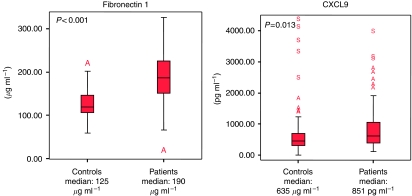
Serum concentration of CXCL9 and Fibronectin 1 in cancer *vs* normal volunteers.

**Figure 3:**
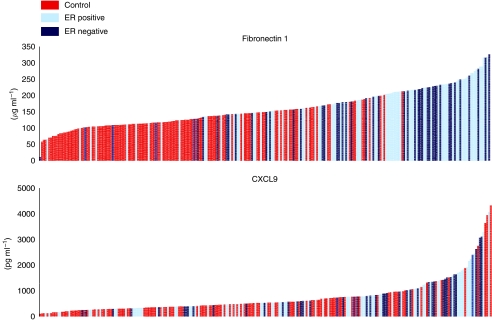
CXCL9 and Fibronectin 1 serum concentrations according to cancer status and ER expression.

Fibronectin 1 concentration was not associated with any clinicopathological characteristics in cancer patients, except age (*P*<0.001; Table 2). As reported in Figure 2, the serum concentration of Fibronectin 1 was higher in cancer patients (mean: 190, min: 11, max: 326 *μ*g ml^−1^) compared with normal volunteers (mean: 125, min: 58, max: 212 *μ*g ml^−1^) (*P*<0.001). Fibronectin 1 concentrations according to cancer status and ER expression are reported in Figure 3.

The metric performances of Fibronectin 1 for BC diagnosis are reported in Table 3. Area under the curve was 0.77 when a cutoff of 150 was chosen, with a 75% sensitivity and 80% specificity. When the analysis focused on ER-negative BC, sensitivity was 72% and specificity was 79% (overall accuracy=77%).

When a cutoff of 200 *μ*g ml^−1^ was chosen, the sensitivity and specificity were 43 and 98%, respectively. Using this cutoff, the positive predictive value was 97%.

### CXCL9 serum concentration in cancer patients *vs* normal volunteers

The CXCL9 serum concentrations according to clinical characteristics are reported in Table 2. The average CXCL9 serum concentrations were 999 and 773 in patients with ER-negative and -positive disease (*P*=0.07, *t*-test), data consistent with gene expression data. As reported in Figure 2, the serum concentration of CXCL9 was higher in cancer patients (mean: 851, min: 121, max: 3941 pg ml^−1^) compared with normal volunteers (mean: 635, min: 12, max: 4327 pg ml^−1^) (*P*=0.013). When the analyses focused on ER-negative disease, the difference between BC samples (mean concentration: 999) and normal volunteers (mean concentration: 635) was statistically significant (*P*=0.003, *t*-test). At the opposite end, CXCL9 concentrations were not different between ER-positive BC (mean concentration: 773) and normal volunteers (mean concentration: 635) (*P*=0.14). CXCL9 concentrations according to cancer status and ER expression are reported in Figure 3.

The metric performances of CXCL9 for BC diagnosis are reported in Table 3. Area under the curve was 0.624 when a cutoff of 750 pg ml^−1^ was chosen, with a sensitivity of 45% and specificity of 79%. When the analyses focused on ER-negative disease, using the same cutoff at 750 pg ml^−1^, sensitivity and specificity were 57 and 80% respectively. Overall accuracy was 73% for the detection of ER-negative BC.

When a cutoff of 1000 pg ml^−1^ was chosen, sensitivity was 27% and specificity was 90%. Using this cutoff, the positive predictive value was 76%.

### Combined detection of FN1 and CXCL9 for breast cancer detection

As stated in the beginning, the objective of serum marker for BC screening would be to increase the performance of a mammogram. Overall, the screening test is expected to be a combination between a serum marker and a mammogram. The goal would be to detect an optimal rate of BC (including ER-negative BC) while keeping a maximal positive predictive value and specificity to avoid unnecessary exams for false-positive results. We considered cases with Fibronectin 1<150 pg ml^−1^ as negative and those with Fibronectin 1>200 pg ml^−1^ as positive. For intermediate concentrations (150<Fibronectin 1>200 pg ml^−1^), patients with CXCL9 concentrations >1000 pg ml^−1^ were considered as positive, and those with CXCL9<1000 pg ml^−1^ were considered as negative. Using this algorithm, we found that 73 and 177 cases were positive and negative respectively. The sensitivity to detect cancer was 53% and specificity was 97%. The positive predictive value was 96%. This score allowed detection in 25 out of 46 ER-negative BCs (54%).

## Discussion

In this study, we have reported that CXCL9 and Fibronectin 1 serum concentrations are higher in BC patients compared with normal volunteers. CXCL9 is an interferon-induced chemokine, involved in T-cell attraction (Liao *et al*, 1995; Gasperini *et al*, 1999). Several studies have shown that CXCL9 is released by cancer cells, including melanoma (Kunz *et al*, 1999) or renal cancer cell (Bukowski *et al*, 1999). Fibronectin 1 is a glycoprotein involved in cell–matrix and cell–cell adhesion, cell migration and oncogene transformation (Gould *et al*, 1990), as well as in tumour invasion and metastasis (Yamada *et al*, 1985; Schwartzbauer, 1988; Humphries and Yasuda, 1988; Couchman *et al*, 1990).

The two proteins, Fibronectin 1 and CXCL9, were identified by the analysis of differentially expressed genes between malignant and benign lesions of the breast. This strategy could be extended for other frequent tumour types, including lung cancer (Humphries and Yasuda, 1988), for which new screening tools are needed. Optimal BC screening is expected to decrease tumour size and lymph node involvement at diagnosis. Unfortunately, BC screening by mammogram misses a significant rate of BC (interval cancer) and is associated with a high rate of false-positive results, requiring unnecessary biopsies. This latter point is particularly true for new technologies including MRI. With the current cutoff, the present serum marker exhibits average sensitivity but high specificity and positive predictive value. When combined with a mammogram, this test is expected to improve the detection of BC modestly. The resulting performance will be an increased sensitivity for the screening test. If developed in combination with a mammogram, it is important for the test to present a high specificity to avoid additional false-positive results to the ones of mammogram. Also, with a high specificity biopsy could be avoided and surgery be performed in patients with ACR4/5 lesion who present a positive serum screening. Finally, adding a serum test with high negative predictive value to mammogram could also improve patient care by avoiding unnecessary biopsy in patients with ACR4 mammograms. This test presents only modest negative predictive values and will not avoid biopsies in these latter patients.

Integration of the Fibronectin 1/CXCL9 score in a screening and post-screening programme could allow both to increase the rate of detected cases and to decrease the rate of unnecessary biopsies. The Fibronectin 1/CXCL9 score looks particularly interesting to detect ER-negative BCs (54% detection). Mass screening is associated with poor detection of these aggressive BCs. In a recent study (Yang *et al*, 2008), it was suggested that for triple-negative BC, mammogram may not be the ideal imaging technique. This is related to both the radiological (lack of microcalcification, occurrence in high-density BC) and the biological (high proliferation rates) features of such cancers. The present data suggest that Fibronectin 1/CXCL9 score could allow to screen a significant rate of patients with ER-negative BC.

Several studies have evaluated the use of serum biomarkers for BC care. None of these studies could identify a robust marker for BC screening. Some studies have shown that although pre-operative CA15-3 has a prognostic relevance (Shering *et al*, 1998), others do not (Ebeling *et al*, 2002). Some data support that CEA is a strong independent prognostic factor for disease-free survival and death from disease (Ebeling *et al*, 2002). Also, these two markers have been studied in the monitoring of treatment efficacy; however, both were related to be poor prognostic markers for determining progression (Bartsch *et al*, 2006).

Some bioassays have been set up with the aim of screening BC patients. Several reports (Tamkovich *et al*, 2005; Huang *et al*, 2006) have suggested that the concentration of tumoural DNA was higher in the serum of BC patients compared with healthy volunteers. Proteomics is another promising technology for BC screening and diagnosis (Davis and Hanash, 2006). Nevertheless, none of these bioassays has shown enough performance to be transferred onto clinical practice.

Although this study reports two candidate proteins that could screen a significant rate of patients with BC, it presents several limitations. First, no validation set has been included in the analysis. A prospective clinical study is ongoing that will better assess the metric performance of the bioassay. Second, the population of normal volunteers included blood donors who are selected for not presenting any symptom or acute disease. Further validation will need to select negative controls carefully. Finally, the sensitivity of the test needs improvements. Additional proteins will be included in the bioassay. These proteins have been selected to be encoded by genes overexpressed in BC, but for which no ELISA was feasible.

In conclusion, analysis of differential gene expression between cancer and normal tissue allows the identification of candidate proteins for blood-based cancer screening.

## Figures and Tables

**Table 1:** Patient characteristics

**Characteristics**	***n*=133 (100%)**
Median age at diagnosis (range)	47 (32–60)
	
*Clinical tumour size*	
T1	25 (19)
T2	72 (54)
T3-T4	34 (25.5)
Unknown	2 (1.5)
	
*Histology*	
Ductal *in situ*	2 (1.5)
Ductal invasive	101 (76)
Lobular invasive	12 (9)
Other	18 (13.5)
	
*Nodes*	
0	20 (15)
1–3	18 (13.5)
>3	15 (11.5)
Not assessable (preoperative chemotherapy)	80 (60)
	
*ER*	
Positive	85 (64)
Negative	46 (34.5)
Unknown	2 (1.5)
	
*PR*	
Positive	70 (52.5)
Negative	61 (46)
Unknown	2 (1.5)
	
*Her-2 neu*	
Positive	30 (23)
Negative	76 (57)
Unknown	27 (20)
	
*Histological grade*	
I	10 (7.5)
II	64 (48)
III	50 (37.5)
Unknown	9 (7)

Abbreviations: ER=estrogen receptor; PR=progesterone receptor.

**Table 2:** CXCL9 and Fibronectin 1 median serum concentrations according to clinical characteristics

**Characteristics (*n*=133)**	**CXCL9 (pg ml^−1^)**	***P***	**Fibronectin 1 (*μ*g ml^−1^)**	***P***
*Age*				
<35	683	0.27	128	<0.001
>35	788		177	
				
*Clinical tumour size*				
T1	874		206	
T2	791	0.69	186	0.35
T3	953		184	
T4	1003		199	
				
*Histology*				
Ductal *in situ*	766		222	
Ductal invasive	845	0.05	191	0.27
Lobular invasive	593		187	
				
*Nodes*				
0	882		186	
1–3	762	0.35	190	0.78
4–9	1044		196	
>9	711		202	
				
*ER*				
Positive	773	0.07	190	0.89
Negative	999		189	
				
*PR*				
Positive	761	0.09	192	0.54
Negative	957		187	
				
*Her-2 neu*				
Positive	888	0.29	186	0.71
Negative	741		182	
				
*Histological grade*				
I	728	0.21	182	0.62
II	774		187	
III	998		196	

Abbreviations: ER=estrogen receptor; PR=progesterone receptor.

**Table 3:** Metric performance of Fibronectin 1 and CXCL9 dosages for BC diagnosis

	**Sensitivity (%)**	**Specificity (%)**	**Positive predictive value (%)**	**Negative predictive value (%)**	**Accuracy (%)**	**AUC**	**95% CI**	***P***
MIG 750 *μ*g ml^−1^	45	79	71	56	73	0.624	0.55–0.69	0.001
MIG 1000 *μ*g ml^−1^	28	90	76	52	57	0.588	0.51–0.65	0.016
FN1 150 pg ml^−1^	75	80	81	74	77	0.778	0.71–0.83	<0.001
FN1 200 pg ml^−1^	42	98	97	60	41	0.706	0.64–0.70	<0.001

Abbreviation: AUC=area under the curve.
